# Low-Grade Appendiceal Mucinous Neoplasm (LAMN) Primarily Diagnosed as an Ovarian Mucinous Tumor

**DOI:** 10.1155/2021/5523736

**Published:** 2021-04-22

**Authors:** Konstantinos Perivoliotis, Gregory Christodoulidis, Athina A. Samara, Ioanna-Konstantina Sgantzou, Theodoros Floros, Georgios Volakakis, Foteini Karasavvidou, Konstantinos Tepetes

**Affiliations:** ^1^Department of Surgery, University Hospital of Larissa, Greece; ^2^Department of Radiology, University Hospital of Larissa, Greece; ^3^Department of Pathology, University Hospital of Larissa, Greece

## Abstract

**Background:**

Low-grade appendiceal mucinous neoplasms (LAMN) are detected in 0.7 to 1.7% of all appendicectomies. The diagnosis can be challenging, particularly in female patients where the differential diagnosis of primary appendiceal and ovarian mucinous neoplasms is unclear. *Case Presentation*. A 71-year-old female was referred to our tertiary hospital with the working diagnosis of a right ovarian cystic tumor. The lesion was identified through a transvaginal ultrasound performed for vague lower abdominal pain symptoms. CT scan confirmed these findings. Intraoperatively, an appendiceal mucocele was identified and a right hemicolectomy was performed. The histopathology examination revealed a LAMN. Six months later, the patient remains disease-free. A close biannual oncological follow-up has been suggested.

**Conclusion:**

This case underlines the difficulty in determining the origin of mucinous neoplasms of the right pelvic area. Mucocele of the appendix should be considered in the differential diagnosis of a mass in the right iliac fossa.

## 1. Introduction

An appendiceal malignancy can be detected in up to 1% of appendicectomy specimens [1]. Low-grade appendiceal mucinous neoplasms (LAMNs) are among the rarest appendiceal tumors with an incidence approximately 0.7-1.7% [2].

LAMNs are characterized by adenomatous alterations in the appendiceal mucosa with uncertain malignant potential, primarily described as “mucinous cystadenomas” [3]. “Pushing invasion” though the appendiceal wall represents a typical pattern for LAMNs, with an increased possibility of ovarian involvement [1]. Ovarian infiltration in appendiceal mucinous neoplasms is well established, with ovarian metastases found in half of patients with appendiceal tumors [4, 5].

A LAMN can either be an incidental intraoperative finding or present with right lower quadrant symptoms [6]. Symptoms originate, mainly, from the distention of the appendix by intraluminal mucus accumulation, i.e., a mucocele; this represents a rare entity, identified in, approximately, 0.2-0.4% of all resections [7].

The diagnosis can be quite challenging, particularly in female patients where primary appendiceal and ovarian mucinous neoplasms share common atypical clinical and imaging findings [8]. Recent studies assessed the real incidence of primary mucinous ovarian tumors at approximately 3% and suggest the theory that most mucinous ovarian malignancies are metastatic [4].

Herein, we present a rare case of a female patient with an appendiceal mucocele (low-grade appendiceal mucinous neoplasm (LAMN)), with a primary misdiagnosis as an ovarian lesion.

## 2. Case Presentation

A 71-year-old Caucasian female was referred to the Gynecological Department of our tertiary hospital with the diagnosis of a right ovarian cystic tumor. The lesion was identified through a transvaginal ultrasound performed for vague lower abdominal pain symptoms that had started a month prior. Findings from the routine laboratory examinations were unremarkable, and all tumor markers were within normal range. The patient had received a screening colonoscopy six months prior without any pathologic findings. Her medical history included hypertension, hypothyroidism, and depression.

**Figure 1:**
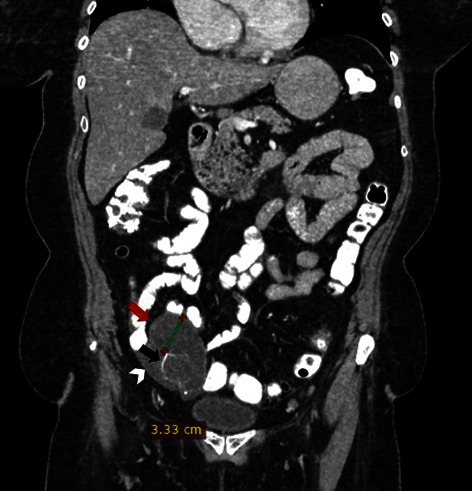
Coronal CT: located in the anatomic region of the right iliac fossa, a low-attenuation cystic lesion that measures 3.3 cm × 6.5 cm with asymmetric wall thickening (red arrow) and focal calcification (black arrow). Peritumoral ascitic fluid can be detected (white arrow).

A Multidetector Computer Tomography (MDCT) was conducted for further evaluation of the findings. A low-attenuation cystic lesion, with asymmetric wall thickening and focal calcification, was identified in the anatomic region of the right iliac fossa, measuring 3.3 × 6.5 cm. CT was in concordance with the previous reports and confirmed that the tumor originated from the right ovary. Peritumoral ascitic fluid was also detected. Distal metastases or pathological regional lymph nodes were not identified (Figure 1).

Thus, the patient was scheduled for surgical treatment. Intraoperatively, with a midline subumbilical incision, a distended appendix, with an intact thick wall and without any inflammatory signs, was identified. Macroscopically, there were no signs of distal metastasis or tumor deposits. Additionally, no pathological findings were identified in the ovary. Following the expansion to a midline incision for better visualization, a decision was made to perform a right hemicolectomy, and the tumor was excised unruptured. The patient had an uneventful recovery; oral feeding began on the 3^rd^ postoperative day, and the patient was discharged on the 9^th^ postoperative day.

**Figure 2:**
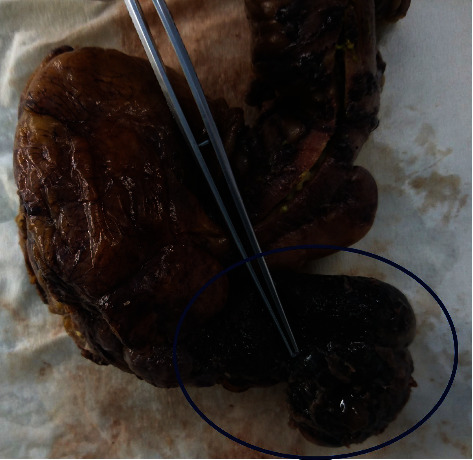
Gross specimen showing the dilated appendix.

**Figure 3:**
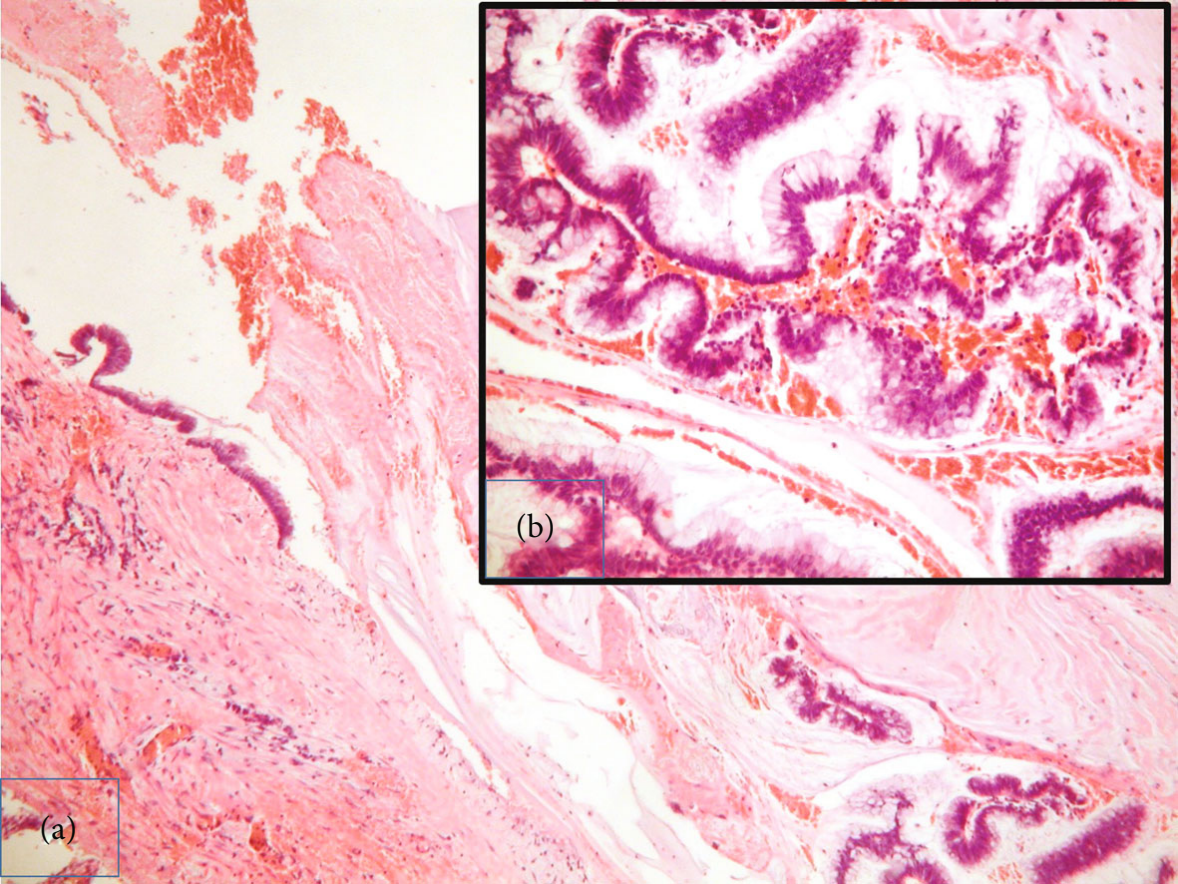
(a) Histopathology of low-grade mucinous neoplasm of the appendix characterized by flat mucinous epithelial proliferation replacing the mucosa and fibrosis of the wall. H/E ×400. (b) Mucin-producing columnar glandular epithelium with low-grade dysplasia H/E ×200.

Macroscopic examination of the specimen revealed a gross dilatation of the appendix measuring 6.5 × 4.5 × 4 cm with abundant mucin in the lumen (Figure 2). Normal morphological characteristics of an appendix were not recognized (Figure 3). Microscopic examination showed replacement of the normal appendiceal epithelium by mucin-producing columnar glandular epithelium with low-grade dysplasia. The lining was flat with few areas of villous architecture. There was fibrosis of the underline wall with scattered residual smooth muscle fibers throughout the caecal wall (Figure 3). There were no histological findings of conventional, high-grade mucinous appendiceal adenocarcinoma, such as proliferation of mucinous epithelial cells with high-grade dysplasia, architectural complexity, cribriform pattern, or glands infiltrating in a desmoplastic stroma.

Based on these, a multidisciplinary team suggested that there was no need for adjuvant chemotherapy and the patient was introduced to a close follow-up schedule. Six months later, the patient remains disease-free.

## 3. Discussion

Mucinous tumors of the appendix are a heterogeneous group of diseases with varying malignant potential [3]. The histopathological classification and diagnosis are not always clear, and terminology to be used has only recently been determined [9]. In 2016, a consensus regarding the classification of pseudomyxoma peritonei and associated appendiceal neoplasia replaced the term “mucinous cystadenomas” with the new term “low-grade appendiceal mucinous neoplasm—LAMN” [3]. In addition, the term “high-grade appendiceal mucinous neoplasm—HAMN” was suggested to describe noninfiltrative invasion lesions with high-grade cytologic atypia [10].

In adult patients, older than 40 years old, the incidence of an appendicular neoplasm complicated with an acute inflammation (appendicitis) ranges between 3% and 17%. For the patients who receive a conservative treatment, the World Society of Emergency Surgery guidelines suggest a close follow-up with colonoscopy and contrast-enhanced CT scan, as appendicitis could harbor a tumor and nonoperative management increases the risk of an underlying lesion misdiagnosis [11, 12]. In such a case, an initially curable neoplasm may progress to an advanced, metastatic, or inoperable malignancy with detrimental survival effects.

When surgical management is decided, a laparoscopic approach (single-port or multiport) should be considered in experienced centers as it offers better visualization of the peritoneum cavity [13, 14]. Despite often presenting as complicated appendicitis, the application of the minimal invasive principles in the treatment of LAMNs results to improved cosmesis and enhanced postoperative recovery, with no effect on the oncological radicality [13, 14].

On the contrary, mucinous ovarian tumors have a reported incidence between 7% and 14% [8]. It is common in patients with a LAMN to be misdiagnosed with a tumor originating from the right ovary. A simple method to intraoperatively distinguish primary and metastatic mucinous ovarian tumors is based on macroscopic characteristics [15]. However, this can be misleading in advanced and metastatic tumors since the anatomic markings can be infiltrated.

The confirmation of the site of origin is critical for the therapeutic algorithm. Presenting symptoms of LAMN and ovarian mucinous tumors can be overlapping, thus increasing the risk for a misdiagnosis. Among them, lower abdominal pain, nausea, changes in bowel habits, and weight loss are the most common [16].

Gastrointestinal (GI) endoscopy provides little diagnostic information for appendiceal lesions [8, 17]. Although quite infrequent, the volcano sign is a typical endoscopic finding that indicates the appendiceal malignancy diagnosis [18]. Zhang et al. reported that in almost 60% of the patients who underwent a preoperative endoscopy for appendiceal mucinous neoplasms, there was no evidence of malignancy [8]. Similar were the findings in our case, where the patient had a normal colonoscopy 6 months preoperatively.

The lack of specific tumor biomarkers consists an additional impediment in the diagnosis of a primary mucinous appendiceal tumor. Commonly used tumor markers include CEA, CA19-9, and CA125. In a study by Zhang et al., tumor makers were elevated preoperatively in most cases, a possible result of peritoneal dissemination [8]. Furthermore, most of the published reports use CA19-9 and CA125 as a recurrence predictive indicator, rather than a diagnostic biomarker [19].

An appendiceal mucinous neoplasm may be identified as a cystic lesion with internal concentric echogenic layers in the right lower quadrant. In females, identification of the right ovary separate from the mass is essential for a differential diagnosis [17]. Both LAMNs and mucinous ovarian tumors have nonspecific imaging findings. In cases where the appendix is located in or extends to the pelvis, the LAMN may be misdiagnosed as a lesion of ovarian or salpingeal origin [20]. Mucinous ovarian tumors are depicted in MDCT as a cystic mass with density (HU) depending on the composition of mucus. Further imaging findings include a thin wall and, in some cases, linear calcifications. A more specific sign is the stained-glass appearance, due to multilocular and varying density content [21].

It is apparent that the differential diagnosis between an appendiceal and an ovarian mucinous tumor is considerably difficult, specifically in complex and advanced cases. Despite its low incidence, intraoperative findings like large size, unilaterality, expansive smooth mass, and lack of extraovarian involvement are suggestive of a primary ovarian mucinous lesion [22]. The role of the tumor size and laterality in the differential diagnosis has been, also, confirmed in the study by Yemelyanova et al. [4]. The diagnostic perplexity between the two pathologies, also, extends in the histopathology setting. Although CK7, CK20, and CDX-2 immunohistochemical staining is commonly used, the exact localization of the tumoral cell origin is not always conclusive, thus requiring correlation with the clinical findings [22].

A perforated appendiceal mucinous neoplasm can lead to progressive accumulation of mucus in the peritoneal cavity, resulting to pseudomyxoma peritonei (PMP) [23]. Correspondingly, ovarian primary mucinous malignancies have been associated with PMP, with an incidence of up to 14% [8]. Recent studies assessed the real incidence of primary mucinous ovarian tumors at approximately 3% and suggest the theory that most mucinous ovarian malignancies are metastatic from another primary site [4]. Ovarian infiltration in appendiceal mucinous neoplasms is well established, with ovarian metastases found in almost half of patients with appendiceal tumors, whereas 18.2% of patients with macroscopically normal ovaries have microscopic disease [4, 5]. In our case, the appendix was unruptured and no deposit was identified in the ovarian parenchyma.

There is no consensus on the optimal treatment of appendiceal mucocele regarding the type and approach of surgery. Appendicectomy and right hemicolectomy have been proposed for cases of an unruptured appendix, while Cytoreductive Surgery (CRS) and Hyperthermic Intraperitoneal Chemotherapy (HIPEC) are proposed in ruptured cases with PMP [24]. A laparoscopic approach is considered to increase the risk of intraoperative rupture and mucus spread [24, 25]. Overall, the 5-year recurrence rate of LAMN has been estimated at 54% following CRS/HIPEC, compared with other epithelial pathologies and given that low-intensity surveillance is recommended [23, 24, 26, 27].

In the present report, we aimed to underline the difficulties in determining the origin of right pelvic mucinous neoplasms. LAMN should be considered in the differential diagnosis of a mass in the right lower abdominal quadrant.

## Data Availability

Data are available upon reasoning requesting.
